# Emotional problems in preadolescents in Norway: the role of gender, ethnic minority status, and home- and school-related hassles

**DOI:** 10.1186/1753-2000-5-37

**Published:** 2011-11-17

**Authors:** Daniele E Alves, Espen Roysamb, Brit Oppedal, Henrik D Zachrisson

**Affiliations:** 1 Division of Mental Health, Norwegian Institute of Public Health, Oslo, Norway; 2 Department of Psychology, University of Oslo, Oslo, Norway; 3 Norwegian Center for Child Behavioral Development, Oslo, Norway

## Abstract

**Background:**

"The gender gap" refers to a lifelong higher rate of emotional problems in girls, as compared to boys, that appears during adolescence. The gender gap is a well-replicated finding among older adolescents and is assumed to be a cross-cultural phenomenon. However, these cross-cultural studies have not investigated the gender gap in ethnic minorities but sampled ethnic majority adolescents in different countries. Some studies that investigated the gender gap across ethnic groups indirectly (by presenting emotional problem scores stratified by gender and ethnic group) indicate that the gender gap is less prominent or even absent among minorities. The aims of this study were to assess whether the gender gap is found in both majority and minority preadolescents, and to investigate whether a possible (gender and ethnic) group difference can be accounted for by differences in home or school hassles.

**Methods:**

Participants were 902 preadolescent students (aged 10 to 12) from two cities in Norway. We collected self-report measures of emotional problems and home and school hassles. Using mediated moderation analysis we tested whether the interaction effect between gender and ethnic minority background on emotional problems was mediated by home or school hassles.

**Results:**

The gender gap in emotional problems was restricted to ethnic majority preadolescents. School hassles but not home hassles accounted in part for this effect.

**Conclusions:**

The absence of the gender gap among minority as opposed to majority preadolescents may indicate that social circumstances may postpone or hamper the emergence and magnitude of the gender gap in ethnic minority preadolescents. In this study, school hassles partly accounted for the combined gender and ethnic group differences on emotional problems. This indicates that school hassles may play a role in the higher levels of emotional problems in preadolescent minority boys and consequently the absence of a gender gap found in our minority sample.

## Background

Emotional (or internalizing) problems include symptoms of depression, anxiety, and withdrawal and are characterized by intropunitive emotions such as sorrow, guilt, fear, and worry [1]. Emotional problems in preadolescents have serious concurrent consequences; they can, for instance, hamper academic success [2,3] and peer relations [4,5]. The presence of these problems at an early age may also predict higher risk of mental and physical disease in middle age [6,7]. It is therefore important to be able to detect and treat emotional problems as early as possible.

One route to attaining a better understanding of emotional problems is to examine why they are more prominent in some groups than in others. One group that has captured the attention of researchers studying emotional problems is ethnic minorities. Among the hypothesized reasons for higher rates of emotional problems in minority groups are social exclusion and discrimination, and for immigrant minorities, difficulties adapting to the new society of settlement. Nevertheless, findings in the field are inconsistent, and some studies find that minorities are more likely to have emotional problems than ethnic majorities, whereas others find the opposite [8]. Findings can vary across different ethnic minorities living in the same country [9], the same minority group in different countries [10], and depending on whom the informant is [8] and the informant's ethnic background [11]. Moreover, minority versus majority differences in emotional problems have also been found to be different for girls and boys [12].

The literature encompassing gender differences in emotional problems is dominated by studies on depression, both clinical and subclinical. Nevertheless, a well-replicated finding concerning emotional problems in general is that girls in late adolescence are at least twice as likely as boys to become depressed [13-15] or anxious [1], and this gender ratio continues throughout adulthood [1,14]: see Sun [16] for an exception). The term "gender gap" refers to the higher levels of emotional problems in girls as compared to boys [17]. The gender gap has been found repeatedly in samples comprising predominantly ethnic majority adolescents, or in samples in which ethnicity was not specifically investigated [1,13,14,18,19].

Although the gender gap is assumed to be a cross-cultural phenomenon [1,20], the studies that support this finding do not investigate ethnic minorities but (predominantly) ethnic majority adolescents in different countries [20,21]. The term "ethnic minorities" refers to groups that differ from the ethnic group that is considered the norm in a country. In particular, we focus here on minorities whose physical characteristics are distinct from those of the "norm" population and are underrepresented in powerful positions in the majority society. To the best of our knowledge, there is no study directly questioning the existence of the gender gap in ethnic minority samples; in other words, we found no study directly questioning whether the finding that girls have more emotional problems than boys, can be extended to ethnic minority populations. Moreover, there is only one study that implicitly questions the gender gap through findings that compare depressive symptoms across gender and ethnic background [22]. In this study the gender gap is only found in ethnic majority preadolescents. Otherwise, most studies that include both ethnic majority and minority groups while also assessing the effects of gender on emotional problems do not focus on gender differences across ethnic groups but on ethnic differences across gender [10,12,15,23-26]. These studies find that a larger gender gap (i.e., higher levels of emotional problems in girls as compared to boys) seems to be either restricted to or substantially more prominent among adolescents of ethnic majority background [15,22,24-27]; for exceptions, see [26,28]. Studies that only include older minority adolescents (aged 15 and older) suggest that the gender gap may emerge later in ethnic minority adolescents [29,30]. In these older minority samples, the gender gap is replicated with girls reporting higher scores of emotional problems than boys.

The scarce literature on this topic provides limited theoretical accounts as to why the gender difference in emotional problems does not seem to be found as readily in minorities during preadolescence as it is in majority adolescents. However, two studies that investigated emotional problems across gender and ethnic background before age 15 found that minority boys show higher levels of emotional problems than majority boys [22,29]. These studies use this finding to explain the lack of gender differences in minorities. Following this line of thought, high emotional problem scores in girls (minority or not) are expected, while similar scores in boys are unexpected. Moreover, when minority boys' emotional problems are unexpectedly high, the gender gap in the minority group is diminished and sometimes nonexistent. There may be particular social circumstances that inflate minority boys' emotional problems in preadolescence which help explain the later emergence of the gender gap in minorities. Hypothesized risk factors involved may be related to discrimination due to physical and cultural attributes [31], factors related to masculine gender roles in different ethnic groups [8], and problems in the family [32] and at school [22,32,33]. Preadolescent minority boys may not experience some of the social circumstances associated with their "protective" (in terms of emotional problems) gender. Alternatively, minorities may experience hassles that exacerbate emotional problems, and minority boys may be particularly sensitive to some of these hassles. Thus, minorities' levels of emotional problems would be higher than for majority peers, and the differences between minority girls' and boys' levels of emotional problems (i.e., the gender gap) would be smaller. In other words, we could look for clues concerning the gender gap by investigating the "ethnic gap" between minority and majority boys.

As compared to adolescence, findings in preadolescence (i.e., the period between the approximate ages of 9 and 12 [34] are more mixed. Although emotional problems in preadolescence are less common than in adolescents [1], when these problems do occur they should be taken seriously. Compared to externalizing problems (such as hyperactivity and conduct problems), emotional problems are less visible and disruptive to others. It may therefore take longer for the home or school environment to detect and help these preadolescents. Failure to detect emotional problems early can therefore prolong unnecessary suffering.

Among studies investigating only ethnic majority preadolescents, three diverging results emerged: (1) no gender difference in emotional problems before puberty or until age 13 [35,36], (2) boys had more emotional problems than girls [37,38], and (3) girls had more emotional problems than boys [19]. These mixed results show that while the gender gap is a well replicated finding among adolescent majorities, this is not the case among preadolescent majorities. Although some of the mixed results may be due to different measures of emotional problems and/or different levels of pubertal maturity in samples across studies, they do indicate a need for more studies investigating emotional problems in preadolescents.

Some studies suggest that social circumstances may play a role in explaining the gender gap in emotional problems [22,25]. School and home are two central life domains for the socialization of children and adolescents. For children with ethnic minority background the home is the main domain for enculturation (i.e., acquiring of own cultural skills and norms), whereas the school is the main domain for acculturation (i.e., changes resulting from contact with other cultural groups, in this case primarily majority culture) [39]. Majority children have an advantage over minority children, since there is a higher overlap between the rules and codes that they learn in their home environment and those that they learn in the school system.

Thus we aimed to investigate whether hassles related to home and school arenas can account for possible gender and ethnic differences in emotional problems. Moreover, it is important to understand why emotional problems are more common in certain groups. Emotional problems in adolescence have received more focus, and the findings for preadolescence are less conclusive. In light of the scarcity of studies that examine possible gender differences across ethnic minority and majority preadolescents (as opposed to examining ethnic differences across gender), the aim of this study was two-fold: to investigate possible group differences in emotional problems related to both gender and ethnic minority status, and to investigate whether eventual higher levels of emotional problems in particular gender and ethnic minority groups can be explained by (mediated through) home and school hassles.

## Methods

### Data collection

Data for this study was provided by the Youth, Culture and Competence (YCC) study undertaken by the Norwegian Institute of Public Health and approved by the Regional Committee for Medical Research Ethics (REC). The YCC is a longitudinal research program that studies the role of immigration and ethnicity in children's developmental trajectories.

We used first-wave data collected during 2006 and 2007 in two cities in Norway that differ in terms of their immigrant population: Oslo (the capital and home of the country's largest immigrant population, which makes up 27% of the capital's entire population), and Bergen (a city in which the immigrant population is of the same relative size as the country's average: 11%, [40]). Bergen is also similar to the Norwegian average in terms of the relative sizes of what are called Western and non-Western immigrants, whereas the percentage of non-Western immigrants is almost three times as large in Oslo as in Bergen [40]. Children attending grades 5 to 7 (aged 10 to 12 years) in 14 schools (of which 9 were in Oslo) were invited to participate in the study (N = 1603). We selected schools in neighborhoods with a high percentage of immigrant families, because we intended to compare different ethnic minority groups in addition to ethnic Norwegians (majority). Thus, we needed to recruit a substantial number of participants in each ethnic group, and this would be easier to achieve in schools in multi-ethnic neighborhoods.

The children's parents were informed of the YCC through the child's school and asked to provide written informed consent if the child was to participate in the study (in accordance with REC guidelines). In addition, we informed parents of the study through Turkish and Tamil cultural centers in Oslo. We targeted these groups for two reasons: The first is the need for more information on the mental health of children of Turkish and Tamil immigrants in Norway. The second is that these two groups differ in terms of migration motivation: One group comprises mainly labor migrants (Turkey), and the other comprises mainly refugees (ethnic Tamils from Sri Lanka) [41].

The YCC questionnaire was completed by the participants in their respective classrooms during two school hours. Of those recruited through cultural centers, 18 participants with Turkish background (40% of Turkish sample) and 4 with Tamil background (9% of Tamil sample) completed the questionnaire in their respective cultural centers. Research assistants were available if needed during data collection. Of the children invited, 1,042 children participated in the YCC, yielding a participation rate of 65%.

### Identifying minority and majority groups

For ethical reasons, we were not allowed by the REC to directly ask participants about their ethnic background in the questionnaire. Thus, we relied on parental and grandparental place of birth in order to categorize participants according to national background. In the first phase of categorizing the participants, we grouped participants into three broad categories, which we labeled "ethnic status": (1) ethnic minority background (n = 473, parents born abroad and at least 3 grandparents born abroad), (2) ethnic majority background (n = 476, parents born in Norway and at least 3 grandparents born in Norway), and (3) double ethnic status (n = 91, one parent born in Norway and one parent born abroad). Participants with double ethnic status were excluded from further analyses, because their complex mix of majority and specific minority backgrounds required special attention that exceeded the scope of this study.

In the second phase of categorizing the participants, we grouped ethnic minority children into national groups according to maternal place of birth (there were a few participants whose parents were born in two different countries outside of Norway). The result was a broad, although scattered representation of a total of 49 national backgrounds. The only countries containing more than 5% of the sample were: Norway (n = 485), Pakistan (n = 126), Turkey (n = 45), and Sri Lanka (n = 43). Thus, the ethnic minority groups were too small to test for the mediation of hassles on emotional problems across gender and specific ethnic background.

In the third phase of categorizing the participants, we divided the ethnic minority group into two main groups, according to a distinction used by Statistics Norway: (1) a group originating from the European Union or European Economic Area, the United States, Canada, Australia, and New Zealand, and (2) a group originating from European countries outside of the European Union, Asia, Africa, Latin-America, and countries in Oceania other than Australia and New Zealand [41,42]. Since there were only 16 participants in group 1, they were excluded from analysis.

Thus, ethnic minorities in this sample consist of preadolescents whose parents originate from European countries outside of the European Union or European Economic Area, the United States, Canada, Australia, and New Zealand, and who have at least 3 grandparents born abroad.

### Sample

A total of 902 participants met the criteria for inclusion in the sample and were assigned to two categories of ethnic status: either ethnic minority or majority. As Table 1 shows, boys and girls were equally distributed in the sample. In terms of grade attendance, the percentages were 30% in grade 5, 37% in grade 6, and 33% in grade 7. Seventy-nine percent of the sample was from Oslo.

**Table 1:** Correlations, and percentage (categorical variables) or means, standard deviations and range (continuous variables), *N *= 902.

Variable	%/M (SD) [Range]	2	3	4	5	6	7	8	9	10
1. Emotional problems	3.1 (2.3) [0-10]	.38**	.29**	-.19**	-.08*	.13**	.13**	.03	.08*	-.10**

2. School hassles	2.9 (2.3) [0-15]		.41**	-.09**	.04	.20**	.00	-.08*	.07*	.00

3. Home hassles	3.4 (3) [0-27]			.04	.10**	.36**	-.07*	-.03	.00	.03

4. Ethnic status (majority = 1)	53%				.01	.02	-.35**	.02	-.05	.03

5. Gender (boys = 1)	50%					.00	.01	-.03	.05	-.03

6. Perceived economic hardship	1.29 (.45) [2-10]						-.07*	-.06	.06	.00

7. City (Oslo = 1)	79%							.11**	-.05	-.05

8. Fifth grade (5^th ^grade = 1)	30%								-.50**	-.46**

9. Sixth grade (6^th ^grade = 1)	37%									-.54**

10. Seventh grade (7^th ^grade = 1)	33%									

*Note*: Percentages (%) for categorical variables; and means (M), standard deviations (SD) and ranges for continuous variables. *p < .05. ** p < .01. (two-tailed)

Otherwise, 47% of the sample had an ethnic minority background, meaning that participants with an immigrant background were overrepresented in line with the recruitment strategy of YCC. Thus, the sample is not representative of the Norwegian population. However, lack of representativeness does not weaken the associations found in this study. The sample reflects the efforts of the YCC team to recruit participants with an immigrant background, even though the study of specific immigrant groups was not possible in this particular analysis. Among participants with an immigrant background, 70% were Norwegian-born. Among those born abroad, 24% had lived their whole lives in Norway (i.e., parents were temporarily abroad at the time of the participant's birth, or moved to Norway shortly after the participant's birth), and the rest of the group had a mean length of residence in Norway of 6 years.

### Measures

*Emotional problems *were measured by the Norwegian self-report version of the emotional problems subscale of the Strengths and Difficulties Questionnaire (SDQ-S) [43]. The emotional problems subscale consists of five items: "I get a lot of headaches, stomach aches or sickness", "I worry a lot", "I am often unhappy, depressed or tearful", "I am nervous in new situations. I easily lose confidence", and "I have many fears, I am easily scared". Each item is rated "not true" (rated 0), "somewhat true" (rated 1) or "certainly true" (rated 2), and a sum score ranging from 0 to 10 is computed. We used the standards from a large Norwegian study, which designate the range of emotional problem scores from 0 to 4 as low risk and present mean scores of 2.2 (SD = 1.9) for boys and 3.0 (SD = 2.2) for girls [19]. The SDQ has adequate psychometric properties [43] that have been replicated in Norway [19,44,45]. The SDQ-S has been used with different ethnic groups, including Norwegians and mixed ethnicity samples [29,45-48]. In this sample, the emotional problems scale demonstrated satisfactory reliability (α = .68).

Both *home and school hassles *were measured with the question: "How often in the last year did you experience" specific hassles [25]. There were nine specific home hassles and five school hassles. Home hassles were: "My parents are away from home a lot (because of work or other activity)", "I have too much responsibility at home (for smaller siblings, housework, or other activity)", "I hear my parents argue", "My parents fight with each other", "Worries because someone in my family drinks too much alcohol", "Worries because someone in my family is sad or frustrated", "Arguments or conflicts with Mom or Dad", "Worries because one of my siblings are in deep trouble", and "Problems because my parents are much more strict than other parents" (α = .69). School hassles were: " I am afraid of not being smart enough at school", "Big problems in understanding the teacher when he/she is teaching", "Big pressure from those around me to succeed and do well at school", "Problems with one or more teachers", "Arguments or problems with other(s) in class" (α = .57). Each question was rated on a scale ranging from 0 ("no, never") to 3 ("yes, very often"), and summed scale scores were computed.

*Economic hardship *was measured with two items from the scale "Adolescent Perceptions of Family Hardship" [49]. The items were: "How often do your parents argue with each other about not having enough money?", and "How often do you argue with your parents about not having enough money?" (α = .48). They were rated on a scale ranging from 1 ("never") to 5 ("always").

### Missing values

We excluded participants with more than 7 missing items (27% of total items), keeping 99% of the sample (n = 889). We used an expectation-maximization algorithm to impute missing responses for the total of 26 items that were included in the analyses. The dependent variable was approximately normally distributed (skewness = .59, kurtosis problems = -.27), and values for home hassles (skewness = 1.05, kurtosis = 1.28) and school hassles (skewness = 1.37, kurtosis = 2.37) fell within acceptable values.

### Statistical analyses

The level of significance was set at .05. We used SPSS version 17 [50] to conduct hierarchical regression to test the interaction effect of gender * ethnic status on emotional problems, adjusting for the following confounding effects: living in the capital, economic hardship (a proxy for socioeconomic status), and school grade (a proxy for age). These variables were controlled for because they have previously been associated with emotional problems [1,8,24]. The following categorical variables were dichotomized as: gender (0 = girls, 1 = boys), ethnic status (0 = minority, 1 = majority), city (0 = Bergen, 1 = Oslo), and school grade 5 (0 = 6^th ^and 7^th ^grade, 1 = 5^th ^grade), school grade 6 (0 = 5^th ^and 7^th ^grade, 1 = 6^th ^grade), and school grade 7 (0 = 5^th ^and 6^th ^grade, 1 = 7^th ^grade). By dichotomizing gender and ethnic status as explained above, the gender * ethnic status interaction yielded the following groups (0 = minority boys, minority girls and majority girls; 1 = majority boys). This meant that we could compare majority boys with the remaining gender/ethnic status groups, but we could also infer information about the other groups by inverting the direction of the regression coefficients. We conducted an ANOVA to test if there were significant differences in emotional problems among the four groups, i.e. minority boys and girls and majority boys and girls.

We also tested whether the gender * ethnic status interaction effect on emotional problems could be mediated through home or school hassles. For this, we used a mediated moderation model [51]. This model tests whether the interaction effect of gender and ethnic status on emotional problems is possibly mediated through home or school hassles. For example, with the variables in our study a mediated moderation model is supported if the following conditions are met: (1) the effect of gender * ethnic status on the mediator (i.e., school/home hassles) is significant, (2) the effect of gender *ethnic status on the outcome variable (i.e., emotional problems) is significant, (3) the proposed mediator (school/home hassles) has a significant effect on the outcome variable after controlling for the interaction effect, and (4) the interaction effect on the outcome is substantially reduced after controlling for the mediator. Thus, mediated moderation is when the initial variable (an interaction) affects the outcome in the first condition (when the mediator is the outcome) and has a weaker effect on the outcome in the second condition (when the dependent variable is the outcome) [51]. Translating this to our analyses, we have mediated moderation when the interaction of gender * ethnic status on emotional problems is weaker after introducing school hassles to the regression.

## Results

We compared mean scores for emotional problems across the four groups of ethnic minority boys and girls, and ethnic majority boys and girls. An ANOVA found significant differences in emotional problems between groups, F (1, 894) = 33.72, *p *= .000 Tuckey's post-hoc test showed that majority boys reported significantly fewer emotional problems than the other groups. Mean scores of emotional problems were identical for minority girls 3.6 (SD = 2.4) and boys 3.6 (SD = 2.4), but they differed significantly for majority girls 3.0 (SD = 2.2) and boys 2.4 (SD = 2.1), *p *< 0.01. When comparing emotional problems within gender, the magnitude of the difference between minority and majority boys (eta square = .07) was more than 3 times the difference between minority and majority girls (eta square = .02). The results for majority boys and girls were similar to those in a large Norwegian study (no specific results for minority groups were reported [19]). Descriptives and intercorrelations are presented in Table 1. As Table 1 shows, emotional problems were correlated with the following characteristics: being a girl, being an ethnic minority, living in the capital, attending grade 6, not attending grade 7. No correlation was found for attending grade 5. The correlations between different grades and emotional problems were unexpected, since increasing age in preadolescence is associated with higher levels of emotional problems [1]. In addition, emotional problems were associated with economic hardship, school hassles, and home hassles.

Next, we proceeded to test a mediated moderation model [51] to test if the interaction effect of ethnic status and gender on emotional problems was mediated by hassles (see "Statistical Analyses" for details on the conditions that support a mediated moderation model). In order to examine whether school or home hassles could explain the low levels of emotional problems in majority boys, we tested a mediated moderation model with the covariates economic hardship (proxy for SES) and dummy variables for city and school grade (proxy for age), since we wanted to control for these factors. As shown in Table 1 economic hardship and city were correlated with emotional problems. We also controlled for school grade since we used it as a proxy for age, and since increasing age in late preadolescence/adolescence is correlated with emotional problems.

As Table 2 shows, school hassles was the only proposed mediator that met the first condition of the mediated moderation model (see 'Statistical analyses' in the Methods section). In other words, the gender * ethnic status interaction significantly affected one of the hypothesized mediators, school hassles (β = -.16, *p *< .005), whereas there was no such effect on home hassles (β = -.06, *p *= .29). This meant that majority boys reported fewer school hassles but not home hassles than the other three gender/ethnic status subgroups. Perceived economic hardship was the only control variable that had a direct effect on school hassles (β = .20, *p *< .005). Belonging to the ethnic majority (i.e., ethnic status = 1) had a small direct negative effect on school hassles (β = -.09, *p *< .05), whereas no such direct effect was found for gender (β = .04, *p *= .28). Since only school hassles met the first condition of the mediated moderation model, home hassles were not included in further analyses.

**Table 2:** Regression analyses: Effect of gender * ethnic status interaction on home and school hassles, controlling for covariates

Predictor	B	SE B	β	R^2^
***DV = School Hassles***				

***Step 1***				

Economic hardship	1.05	.17	.20***	.05***
City	.15	.19	.03	
5^th ^grade	-.27	.20	-.05	
6^th ^grade	.18	.18	.04	

***Step 2***				.06*

Gender	.17	.15	.04	
Ethnic status	-.41	.17	-.09**	

***Step 3***				.06***

Gender * ethnic status	-.86	.31	-.16***	

***DV = Home Hassles***				

***Step 1***				

Economic hardship	2.32	.21	.35***	.13***
City	-.34	.23	-.05	
5^th ^grade	-.10	.24	-.02	
6^th ^grade	-.19	.22	-.03	

***Step 2***				.14**

Gender	.57	.19	.10***	
Ethnic status	.11	.20	.02	.29

***Step 3***				

Gender * ethnic status	-.39	.37	-.06	

*Note: *DV = dependent variable; B = unstandardized regression coefficient; SE B = standard error of the regression coefficient; β = stardardized coefficients; R^2 ^= coefficient of determination.

*p < .05. **p < .01. ***p < .005.

**Figure 1:**
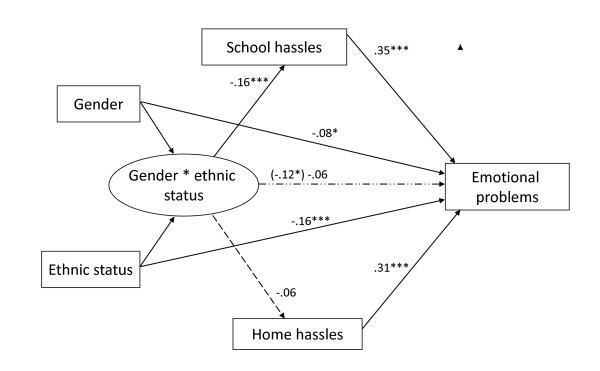
**Mediated Moderation Model**. Numbers represent standardized regression coefficients. (_____) significant paths; (— — —) non-significant paths; (— . . —) path from gender*ethnic status to emotional problems is significant before controlling for school hassles. *P < .05. **P < .01. ***P < .001.

As Table 3 shows, the second condition was also met, as the interaction effect of gender * ethnic status on emotional problems was significant (β = -.12, *p *< .05; see Step 3a). The third condition was met, as school hassles had an effect on emotional problems after the effect of the gender * ethnic status interaction was controlled for (β = .35, *p *< .005; see Step 3b). The fourth and last condition of the mediated moderation model was met as the effect of the gender * ethnic status interaction was substantially reduced after controlling for school hassles (β = -.06, *p *= .29, see Step 3b). The Sobel test [51] was used to statistically investigate the effect of the hypothesized mediator (i.e., school hassles) on the predictor-outcome (i.e., gender * ethnic status-emotional problems) relationship. Results of the Sobel test indicated that school hassles significantly mediated the influence of gender * ethnic status on emotional problems (*z *= -2.70, *p *< .01). Since all conditions were met, we concluded that the proposed mediated moderation model was supported. In other words, the above analyses suggest that the effect of the gender * ethnic status interaction in predicting emotional problems partially is mediated through school hassles (see Table 3). Figure 1 is a conceptual model that summarizes the central relationships in the mediated moderation model (control variables not included and note that school and home hassles were not entered simultaneously in the analyses.). Figure 1 shows that the effect of gender * ethnic status (the predictor) on emotional problems (the outcome) decreases from β = -.12 to β = -.06 when school hassles (the mediator) is included in the regression analysis. In other words, low emotional problems in majority boys (by dichotomization: gender = 1 * ethnic status = 1) are in part explained by low levels of school hassles. Alternatively, high emotional problems in the other three gender/ethnic status groups (among others minority boys, who according to their gender would be expected to show lower emotional problems) are, in part, explained by high levels of school hassles.

**Table 3:** Regression analysis: Testing the mediated moderation model

Predictor	B	SE B	β	R^2^
***Step 1***				.05***
SES	.14	.03	.14***	
City	.15	.04	.13***	
5^th ^grade	.08	.04	.08*	
6^th ^grade	.11	.04	.11***	

***Step 2***				.08***

Gender	-.07	.03	-.08*	
Ethnic status	-.15	.03	-.16***	

***Step 3a***				.08*

Gender * ethnic status	-.60	.30	-.12*	

Mediator: School Hassles				

***Step 3b***				.20***

Gender * ethnic status	-.30	.28	-.06	
School hassles	.35	.03	.35***	

*Note: *DV = dependent variable; B = unstandardized regression coefficient; SE B = standard error of the regression coefficient; β = stardardized coefficients; R^2 ^= coefficient of determination. *p < .05. **p < .01. ***p < .005.

## Discussion

In this study the gender gap in emotional problems was found in preadolescents with ethnic majority background, whereas differences were absent in minority preadolescents. Compared to normative levels of emotional problems in the Norwegian population [19], minority preadolescents in this study report higher scores. The difference was particularly salient among boys, although the effect size was small and clinically insignificant. We also found that school hassles, but not home hassles, accounted for the combined gender and ethnic status differences found in the sample. This means that low levels of school hassles among majority boys in part accounted for their low levels of emotional problems, as compared to the three other gender/ethnic status groups.

For majority children, the results replicate previous findings indicating a gender gap in emotional problems in adolescence (sometimes already during preadolescence, as in this study). However, this finding was not supported in minority preadolescents. That is, no gender differences in emotional problems were found for minority preadolescents. The explanatory effect of minority background on emotional symptoms was only 3%, which is small but significant (See standardized regression coefficient of ethnic status, β = -.16 in Table 3 and square it for information about its contribution to emotional problems). For comparison purposes we may look at gender, which is considered an important variable in the investigation of emotional problems, yet it accounted for 1% of the variance (see Table 3). In comparison, school hassles explained 12% of the variance in emotional problems.

So what seems to support the larger gender gap in emotional problems in ethnic majority as compared to ethnic minority preadolescents? In our study, the subgroup that at first glance most distinguishes itself is majority boys, who have the lowest scores for emotional problems. Furthermore, it seems that one of the "protective factors" for their emotional health is their low reports of school hassles. Alternatively, we could argue that it is not the majority boys that stand out as a group due to low levels of emotional problems but the minority boys for their relatively high levels in spite of their "protective" gender. As mentioned previously, emotional problem scores for majority boys and girls in our study are quite consistent with those in a large Norwegian epidemiologic study [19]. This consistency in the emotional problems of majority boys may indicate that the minority boys are the ones with levels of emotional problems above the expected. However, we should keep in mind that the gender gap may appear later in ethnic minority groups than it does in ethnic majority groups.

In line with findings from Kistner et al. [22], our results point to school-related hassles as potential mediators for minority boys' emotional problems. Steele [52] suggested that members of disadvantaged groups, for whom negative stereotypes of low academic achievement are prominent, are likely to experience distress about confirming the negative stereotype. Applying Steele's suggestion to this study, we could reason that ethnic minority boys may protect their self-esteem by reducing their identification with academic performance [22,53]. However, this strategy may increase emotional problems in the long run, as school hassles accumulate [52]. Nevertheless, it is important to stress the plausibility that different risk factors may be relevant for different ethnic minority groups. For instance, higher levels of emotional problems in boys with Pakistani and Tamil background in Norway may be due to different social circumstances. One hypothesis is that being different from the norm (either physically or culturally) may put preadolescent minority boys at a higher risk of developing emotional problems than what would otherwise be expected in boys. Alternatively, boys from specific minority groups may have different risk factors for developing emotional problems, such as parental expectations concerning academic performance.

A number of limitations apply to this study. All measures were self-report, so we cannot rule out the possibility that preadolescents reported high levels of school hassles because of high levels of emotional problems, and not the other way around, as suggested above. Moreover, this study is cross-sectional and cannot shed light on causality issues. Considering informant variability with measures such as emotional problems, the study would have been strengthened by multiple assessment methods. It is possible that there is gender and culturally related bias in reporting emotional problems. Moreover, the conclusions are generalizable only for emotional problems and not for clinically significant depression or anxiety.

An additional limitation of this study was that we were not able to analyze different ethnic groups within the minority population in Norway. Testing whether school hassles mediate the interaction between gender and ethnic status on emotional problems, while simultaneously controlling for city, economic hardship, and school grade attended, requires a large sample size to gain adequate statistical power. We were therefore unable to investigate distinct ethnic groups as originally planned. Thus, collapsed different groups of non-Western minorities. These groups are undoubtedly quite different, but they share several similarities, such as their status as ethnic minorities; most of them are physically salient, differing from the ethnic majority group. Additionally, their upbringing is more likely to stress collectivistic values than individualistic values (as predominantly endorsed by most ethnic Norwegians). Because the categorization of ethic status groups was based on self-reported parental place of birth, there are two other main misclassification issues: Misclassification of minorities into the majority group and misclassification of majority into minority groups. The first group consists of preadolescents of parents born in Norway, but who have indigenous, religious or minority background and whose parents look different or/and hold different cultural values than those pertaining to the majority. The second group consists of preadolescents of parents born abroad by sojourners, that is, preadolescents of ethnic Norwegians who were born while parents were temporarily abroad or emigrated to another country. However, misclassification was minimized since we also had data on the birthplace of grandparents. In other words, the ethnic status distinction we applied lacked precision, although its use was reasonable, especially given previous findings suggesting that minorities and majorities differ in relation to the gender gap. Ideally, we would have national groups large enough for comparison, and this should be an ambition for future studies. When addressing the effect of ethnic background on emotional problems, additional factors to consider are the "ethnic ratio" of the neighborhood [54] or schools [55] where preadolescents live.

The study also lacks data on pubertal development and acculturation. Pubertal development is associated with gender differences in emotional problems, although it may be a better predictor of emotional problems than chronological age in majority than minority girls [56]. It could be that the ethnic minority groups in the sample reached puberty later than ethnic Norwegians. This would postpone the gender gap in ethnic minorities and could explain why we did not find the gender gap in minority preadolescents. Although interesting, data on pubertal development is not essential to this study since our focus was not on the timing of the gender gap across groups. In terms of acculturation, ethnic minorities who are fluent in Norwegian language and competent in cultural codes (i.e., majority cultural competence) may have lower levels of emotional problems than those who are not as fluent or competent. However, whether majority cultural competence protects the mental health of minorities may depend on their environment. For instance, Dalhaug et al. [55] did not find a positive relationship between majority cultural competence and mental health in schools with a high density of minority adolescents. In the near absence of majority students, knowledge about majority culture and language may not have been crucial to the adolescents' well-being. Thus, where possible, both acculturation and pubertal development should be included in future studies.

This study also has strengths worth noting. It supports that gender differences are found in majority but not in minority preadolescents. Also, in our mediating moderator regression analyses, we were able to study whether differences in emotional problems across gender and ethnic status may be mediated though home and school hassles while simultaneously controlling for important covariates that could confound the associations in the model (economic hardship, school grade attended, and city). Many studies have samples in which these variables are grouped together, and they lose some important information by doing so. In addition, this study supports the role of school hassles as a potentially significant mediator for emotional problems. Future studies should examine the relationship between gender, ethnic background, and emotional problems longitudinally. If a causal link is suggested between school hassles and emotional problems, studies should further investigate what specific types of hassles are associated with these symptoms. The studies could have implications for understanding the mechanisms underlying emotional problems in preadolescence as well as for tailoring interventions to reduce symptoms. Based on our findings, we would particularly suggest that school-related hassles be included in further investigations.

## Competing interests

The authors declare that they have no competing interests.

## Authors' contributions

DEA participated in data collection, conducted literature search and data analyses, and drafted the article. ER made a substantial contribution to the methodology and the interpretation of results and helped draft the manuscript. BO is PI of the YCC project from which data from this study is drawn and helped draft the manuscript. HDZ made a substantial contribution to the design and methodology of the study and the interpretation of results and helped draft the manuscript. All authors read and approved the final manuscript.
